# Retraction: A Comparison of Early Rehabilitation in the Intensive Care Units of Patients With Severe COVID-19: A Propensity Score Matching Analysis

**DOI:** 10.7759/cureus.r70

**Published:** 2023-04-04

**Authors:** Kohji Iwai, Yasuyuki Tsujita

**Affiliations:** 1 Division of Physical Therapy, Faculty of Rehabilitation and Care, Seijoh University, Tokai, JPN; 2 Department of Critical and Intensive Care Medicine, Shiga University of Medical Science, Otsu, JPN

This article has been retracted at the request of the authors due to a miscommunication resulting in the failure to obtain proper ethics review from Shiga University of Medical Science. The authors did obtain ethical review and approval from Seijo University and were under the impression that ethical review from Shiga University of Medical Science was not required. Once informed of their error, the authors contacted the Shiga University of Medical Science ethics committee in an attempt to receive retrospective ethical review and approval, however this request was denied. As a result, the authors have regretfully requested this retraction. After a thorough review, the journal has agreed and the article has been retracted.

